# Longitudinal and cross-sectional analysis of perfluoroalkyl substances and kidney function

**DOI:** 10.1038/s41370-025-00785-z

**Published:** 2025-06-09

**Authors:** A. Eklund, T. Taj, L. Dunder, P. M. Lind, L. Lind, S. Salihovic

**Affiliations:** 1 School of Medical Sciences, Faculty of Medicine and Health, Örebro University, Örebro, Sweden; 2 Clinical Epidemiology and Biostatistics, School of Medical Sciences, Faculty of Medicine and Health, Örebro University, Örebro, Sweden; 3 Department of Medical Sciences, Occupational and Environmental Medicine, Uppsala University, Uppsala, Sweden; 4 Department of Medical Sciences, Uppsala University, Uppsala, Sweden

**Keywords:** PFAS, Perfluorinated chemicals, Kidney function, Glomerular filtration rate, Chemical exposure, Mixed models

## Abstract

**Background:**

Perfluoroalkyl substances (PFAS) constitute a diverse group of chemical compounds used in various consumer products. While the associations between PFAS and certain adverse human health effects are well-documented, their impact on kidney function remains less known.

**Objective:**

The main aim of this study is to investigate the relationship between PFAS levels and kidney function (estimated glomerular filtration rate [eGFR]) utilizing a longitudinal design.

**Methods:**

The population-based Prospective Investigation of the Vasculature in Uppsala Seniors (PIVUS) study included 997 individuals at baseline (all aged 70 years, 50% females). Follow-up investigations were performed at 75 and 80 years of age. Seven major PFAS were determined in plasma using ultra-high performance liquid chromatography-mass spectrometry. Longitudinal and cross-sectional associations between PFAS and eGFR were analyzed using linear regression and mixed effects models following adjustment for sex, HDL and LDL-cholesterol, triglycerides, glucose, BMI, statin use and smoking.

**Results:**

Longitudinal models demonstrated statistically significant positive associations between perfluoroundecanoic acid (PFUnDA), perfluorononanoic acid (PFNA), and perfluorodecanoic acid (PFDA)and eGFR (all *P* < 0.001). The associations between linear perfluorooctane sulfonic acid (PFOS) and perfluorohexanesulfonic acid (PFHxS) followed a similar trend. In contrast, an inverse relationship between perfluoroheptanoic acid (PFHpA) and perfluorooctanesulfonamide (PFOSA) with eGFR was observed. The findings were largely corroborated by cross-sectional analyses.

**Impact statement:**

This longitudinal study found that changes in certain PFAS concentrations were positively associated with the change in kidney function, though the direction of association varied across PFAS. These findings were further supported by cross-sectional analysis. The complexity of associations remains incompletely understood as some PFAS showed positive associations while others were inverse. Further longitudinal studies with repeated measures are needed to better elucidate the relationship between PFAS exposure and kidney function.

## Introduction

Perfluoroalkyl substances (PFAS) constitute a large group of synthetic organic compounds with unique chemical properties. Originating in the 1940s, the chemicals have gained a broad use due to their great stability and thermal resistance attributed to their structure characterized by fluorinated carbon chains [1]. The carbon chain varies in length and functional group attached at the end of the chain, depending on the specific substance (CnF2n + 1−). PFAS have been used in a variety of industries and consumer products including textiles, electronics, medical products and firefighting foams among others [1]. The stability and high mobility of the compounds, however, also makes them persist in the environment and spread to remote regions e.g., the Arctic. The most well characterized PFAS are perfluorooctane sulfonic acid (PFOS) and perfluorooctanoic acid (PFOA).

PFAS are frequently detected in humans and have long elimination half-lives, with an estimated mean half-life of PFOA of 1.5–5.1 years, and PFOS of 3.4–5.7 years [2]. Studies from Europe, the US and China have found detectable levels in over 98% of the samples of general populations [2–5]. Human exposure to PFAS have been primarily attributed to contaminated drinking water and food, but also through inhalation of dust and airborne volatiles [5, 6]. Following entry into the human body, the compounds are distributed in plasma and throughout the body, in organs such as the brain, liver, lung, bone and kidney. As PFAS are not biotransformed in humans, their elimination is dependent on non-metabolic clearance [7, 8] The clearance of PFAS in urine vs feces is dependent on chain length and functional group. Fecal elimination is generally lower than urinary elimination. For PFOA, urinary elimination slightly outweighs fecal elimination, while for PFOS, fecal elimination is significantly higher [9]. These findings highlight the complexity of PFAS elimination and the importance of considering both urinary and fecal routes in toxicokinetic studies.

The evidence of PFAS related adverse effects on human health have been thoroughly reviewed in previous studies [4, 8]. For instance, exposure to the substances have been associated with hyperlipidemia, microvascular disease and the metabolic syndrome, all of which are factors that pose risk for impaired kidney function. Multiple pharmacokinetic and toxicological studies demonstrated several mechanisms that could explain the harmful effects of PFAS. These include oxidative stress pathways, peroxisome proliferator-activated receptor pathways, and NF-E2-related factor 2 pathways.

The kidneys play a crucial role in PFAS elimination, with active proximal tubule transport identified as a key mechanism [7, 10]. Proximal tubule handling of PFAS involves both secretion and reabsorption, mediated by solute carrier protein family transporters, including OAT1 and OAT3 on the basolateral side and OAT4 and URAT1 on the apical side [11]. While GFR partly influences renal excretion by determining the amount of PFAS filtered at the glomerulus, it has been suggested that it does not fully account for variations in PFAS elimination. This is evident from the weak correlation between urine-to-serum PFAS ratios and GFR, suggesting that proximal tubule transport plays a critical, independent role [12]. Factors such as tubular reabsorption and secretion, which may vary due to genetic, epigenetic, or other individual differences, are likely significant determinants of PFAS urinary elimination [12].

Several epidemiological studies have been conducted to investigate the relationship between PFAS and renal function, with most of them adopting a cross-sectional design. Due to the design, reverse causation has been a difficult obstacle in the interpretation of the results. Since PFAS are mainly eliminated by the kidney, a decreased glomerular filtration rate (GFR) may result in slower elimination with resulting increases in serum PFAS over time. However, a few cross-sectional studies have employed methods to limit the risk of reverse causation. Dhingra et al. concluded that kidney function is rather the cause than the result of elevated serum PFOA levels [13]. The study used PFOA concentrations measured in serum and modeled PFOA concentrations based on external exposure estimates which are less susceptible to physiological disturbances and found an association between lower GFR and the measured levels of PFOA, but not the modeled estimates [13]. Contrasting findings were reported in another study that investigated PFOA, PFOS, perfluorohexane sulfonic acid (PFHxS) and perfluorononanoic acid (PFNA), utilizing three different statistical models to evaluate the causal relationship between PFAS and eGFR. The study found that PFAS was associated with a lower eGFR and concluded that the possibility of reverse causation was low in their investigation [14]. The inverse association of PFAS and eGFR has been corroborated by the few existing longitudinal studies [10, 15]. Given the complexity of the relationship between PFAS and kidney function, more longitudinal studies are needed to further validate the findings of previous studies and expand the current knowledge.

The aim of this study is to investigate the relationship between changes in plasma PFAS concentrations and changes in estimated kidney function (eGFR) over 10 years using a longitudinal design. Based on the results from the few previously conducted longitudinal studies our hypothesis was that changes in PFAS levels would be inversely associated with the change in eGFR.

## Materials and methods

### Study population and sample

Longitudinal cohort study including three timepoints spanning over 10 years where the change in PFAS concentration has been previously described [5]. The study population is derived from the epidemiological Prospective Investigation of the Vasculature in Uppsala Seniors (PIVUS) study and has previously been thoroughly well-characterized [16]. In summary, the research sample was randomly selected from the register of community living of Uppsala, Sweden. Each subject obtained an invitation letter within two months of their 70th birthday (between 2001 and 2004). The target cohort consisted of 2025 subjects, of whom 1016 were enrolled (50.1%). A non-responder analysis has been performed, indicating a slightly lower disease susceptibility in the enrolled population. Follow-ups were performed at age 75 (*N* = 814), and at 80 years (*N* = 601), between 2006–2009 and 2011–2014, respectively. Within the present study, 997 participants were included at baseline. A random 19 subjects from the initial 1016 were excluded due to technical issues where samples were missing for PFAS analysis, had insufficient sample volumes, or did not meet the QA/QC criteria following extraction and analysis.

The participants underwent a clinical evaluation which included answering a questionnaire about their medical history, regular prescriptions, smoking habits, and other life-style related aspects. Following an overnight fast, 1–2 mL samples of blood were compiled from each patient in the morning (8–10 am). The blood samples were put in freezers (-70°C) until analyses were performed. Complete information about the study population has been described [16].

### PFAS exposure assessment

We aimed to evaluate the PFAS that showed a measurable level above the lower limit of detection, in at least 75% of the study population at baseline. This included perfluoroheptanoic acid (PFHpA), perfluorooctanoic acid (PFOA), perfluorononanoic acid (PFNA), perfluorodecanoic acid (PFDA), perfluoroundecanoic acid (PFUnDA), linear isomer of PFOS (L-PFOS), perfluorohexane sulfonic acid (PFHxS), and perfluorooctane sulfonamide (PFOSA). PFAS were extracted from 150 microliter samples of plasma or serum using protein precipitation and were filtered through an Ostro 96 well-plate (Waters Corporation, Milford, USA). Subsequently, the PFAS were analyzed in the samples using matrix matched isotope dilution ultra-performance liquid chromatography (UPLC-MS/MS) coupled to tandem mass spectrometry system (Waters Corporation, Milford, USA). Isotope dilution was used to quantify PFAS concentrations, which are stated in ng/ml. The method detection limits for all three investigations ranged from 0.01 to 0.18 ng/ml^−1^ depending on the analyte. The analytical methods employed in the study have been previously developed, validated and thoroughly explained by previously [5, 17]. Values below LOD were replaced by LOD/√2.

### eGFR

To assess kidney function, estimated glomerular filtration rate (eGFR, ml/min/1.73 m^2^) was used. The eGFR was calculated using a validated formula utilizing both cystatin C and plasma creatinine for greater accuracy (CKD-EPI creatinine-cystatin C equation 2012) [18]. Cystatin C and plasma creatinine were measured using a standard enzymatic method and by an enhanced turbidimetric method respectively, prior to incorporation in the following formula:

The CKD-EPI creatinine–cystatin C equation (2012) can be expressed as a single equation: 135 × min(Scr/κ, 1)^α^ × max(Scr/κ, 1)^−0.601^ × min(Scys/0.8, 1)^−0.375^ × max(Scys/0.8, 1)^−0.711^ × 0.995^Age^ [×0.969 if female] [×1.08 if black], where Scr is serum creatinine, Scys is serum cystatin C, κ is 0.7 for females and 0.9 for males, α is −0.248 for females and −0.207 for males, min indicates the minimum of Scr/κ or 1, and max indicates the maximum of Scr/κ or 1.

### Covariates

Selection of covariates used in the statistical analysis were based on evidence presented in previous publications. Sex was included since PFAS concentrations have previously been found to differ between men and women, and to account for sex-associated differences in GFR [19, 20]. Smoking (active; yes or no, %) was included given its association with serum PFAS concentrations and chronic kidney disease [21–23]. Based on previous evidence of independent risk factors of chronic kidney disease, the following covariates were also included in the analysis; serum triglycerides (mmol/l), low-density lipoprotein (LDL)-cholesterol (mmol/l), high-density lipoprotein (HDL)-cholesterol (mmol/l), BMI (kg/m^2^) and blood-glucose (mmol/l) [24]. Statin use (self-reported, %) was included because of their renoprotective effect [25–27]. To prevent interference from drift factors during the sample collection periods, we also included the patients’ recruitment date as a covariate. An overview of the hypothesized causal relationships considered in the present study are provided in a directed acyclic graph (Supplementary Fig. 1).

### Statistical analyses

#### Cross-sectional analysis

To assess the association between PFAS and eGFR at distinct time points, we performed separate cross-sectional analyses at each of the three sampling occasions (age 70, 75, and 80). Linear regression models were used to assess the relationship between eight PFAS (ln-transformed) and eGFR, adjusting for BMI, smoking, cholesterol (HDL and LDL), triglycerides, glucose, and statin use.

#### Longitudinal analysis

To further investigate time-dependent effects, we test both linear and non-linear mixed models to examine the association between PFAS and eGFR over time. To compare the fit of linear versus spline models for the effect of PFAS on eGFR, we used Akaike Information Criterion (AIC) values (Supplementary Table 1). Spline-based models consistently provided a better fit compared to models with linear fit, as indicated by the lower AIC values, and were therefore chosen for the main analysis. To model changes in eGFR over time and account for individual variation, we performed analyses while parameterizing time both as a fixed effect (using linear terms or splines) and as a random effect (random slopes and intercepts) following adjustment for the covariates from Section “Statistical analyses” (age [time], BMI, smoking, cholesterol (HDL and LDL), triglycerides, glucose, and statin use).

The main mixed-effects model can be expressed as:

$${{{{\rm{eGFR}}}}}_{{{{\rm{ij}}}}}= 	 \, {{{{\rm{\beta }}}}}_{0}+{{{{\rm{\beta }}}}}_{1}{{{{\rm{PFAS}}}}}_{{{{\rm{ij}}}}}+{{{{\rm{\beta }}}}}_{2}{{{\rm{ns}}}}({{{{\rm{time}}}}}_{{{{\rm{ij}}}}},2)+{{{{\rm{\beta }}}}}_{3}({{{\rm{ns}}}}({{{{\rm{time}}}}}_{{{{\rm{ij}}}}},2)\times {{{{\rm{PFAS}}}}}_{{{{\rm{ij}}}}})\\ 	 +\sum {{{{\rm{K}}}}}_{{{{\rm{k}}}}=4}{{{{\rm{\beta }}}}}_{{{{\rm{k}}}}}{{{{\rm{Covariates}}}}}_{{{{\rm{ij}}}}}+{{{{\rm{b}}}}}_{0{{{\rm{i}}}}}+{{{{\rm{b}}}}}_{1{{{\rm{i}}}}}{{{{\rm{time}}}}}_{{{{\rm{ij}}}}}+{{{{\rm{\epsilon }}}}}_{{{{\rm{ij}}}}}$$

where natural splines (ns): represent a flexible, piecewise polynomial function of time that allows the effect of time on eGFR to vary non-linearly. Here, ns(time,2) specifies the use of 2 degrees of freedom, which divides the time variable into segments and fits separate curves while ensuring smoothness at the segment boundaries. β_2_ns(time_ij_,2): captures the non-linear main effect of time on eGFR; and β_3_(ns(time_ij_,2) × PFAS_ij_): models the interaction between the time spline and PFAS, allowing the effect of PFAS on eGFR to evolve non-linearly over time.

Missing data were addressed using multiple imputations under the missing at random (MAR) assumption. Five imputed datasets were generated, and the results were pooled using Rubin’s rules to account for uncertainty due to missing data.

Descriptive statistics were reported as means and standard deviations for normally distributed data, percentages for categorical variables or median and interquartile ranges for non-normally distributed data. The beta coefficients (β) and corresponding confidence intervals (CI) are reported on the log-transformed scale, which allows for consistent interpretation of directionality and significance. Statistical significance was set at *p* < 0.05. The statistical analysis was performed using the “mice”, “splines” and “lme4”, packages in R statistical computing software (version 4.2.3; R Foundation for Statistical Computing).

### Sensitivity analysis

To address potential concerns regarding model robustness, we performed several sensitivity analyses. First, to evaluate the potential for over-adjustment, we re-analyzed the models after excluding BMI, HDL, and LDL as covariates (Supplementary Table 2). Second, to assess the possibility of reverse causation, we modeled baseline eGFR as the independent variable to investigate its association with PFAS levels over time (Supplementary Table 3). Finally, as a robustness check, we restricted the analyses to individuals who participated in all three investigations (complete case analysis), thereby ensuring no missing data in the longitudinal analysis (Supplementary Table 4).

### Ethical considerations

The study was approved by the Ethics Committee of the University of Uppsala (Dnr 00419 and 2005/M-079). Participants provided their informed written consent prior to participation and were informed that they could withdraw their consent at any time without facing any consequences. All methods were performed in accordance with the relevant guidelines and regulations.

## Results

### Baseline characteristics of the study participants

The study population comprised 997 individuals at baseline who underwent investigation at three time points spanning the years 2001–2014. Detailed information on the baseline population characteristics is shown in Table 1. The baseline mean age of 70 years, with 50.2% females. Participants had a baseline mean BMI of 27.0 kg/m², and 11% were active smokers. Comorbidities included diabetes (8.7%), myocardial infarction (7.1%), stroke (3.7%), and congestive heart failure (3.8%).

**Table 1:** Baseline characteristics of the study population at age 70 (*N* = 997) reported as means and standard deviation (SD) or percent (%).

Characteristics	Mean +/−SD or %
Age (years)	70.0 +/− 0.2
Females (%)	50.2
Height (cm)	169 +/– 9.1
Weight (kg)	77 +/− 14
Waist circumference (cm)	91 +/− 12
BMI (kg/m^2^)	27.0 +/− 4.3
Waist/hip ratio	0.90 +/− 0.075
SBP (mmHg)	150 +/− 23
DBP (mmHg)	79 +/− 10
Myocardial infarction (%)	7.1
Stroke (%)	3.7
Congestive heart failure (%)	3.8
Diabetes (%)	8.7
Active smoker (%)	11

*BMI* body mass index, *SBP* systolic blood pressure, *DBP* diastolic blood pressure.

Table 2 summarizes the longitudinal trends of change in PFAS levels and change in eGFR across three timepoints spanning 10-years (2001–2014). The longitudinal change in median PFAS concentrations in serum over the 10 years (from 2001 to 2014) has been described previously [5]. The median serum concentrations of PFAS over this period, exhibited non-linear trends. Between baseline and first follow-up, median concentrations of all PFAS, except PFOS and PFOSA, increased. Conversely, between the first and second follow-up, median concentrations of all PFAS decreased. Overall, from baseline to second follow-up, a statistically significant increasing trend in median concentrations was reported for PFUnDA (26%), PFHxS (34%), PFNA (23%) and PFDA (7%). In contrast, statistically significant decreasing trend was observed for median concentrations of PFHpA (−50%), PFOSA (−75%), L-PFOS (−44%) and PFOA (−27%) [5]. eGFR showed a statistically significant and linear decrease during the 10-years.

**Table 2:** Trends of PFAS concentrations, reported as median and interquartile range (IQR), and trends in GFR, reported as mean and standard deviations (SD).

Variables	Age 70 *N* = 997	Age 75 *N* = 814	Age 80 *N* = 601
**PFAS concentration**	**Median (IQR)**	**Median (IQR)**	**Median (IQR)**
PFHpA, ng/ml	0.05 (0.03, 0.09)	0.07 (0.04, 0.11)	0.03 (0.01, 0.06)
PFOA, ng/ml	3.32 (2.54, 4.40)	3.8 (2.71, 5.41)	2.53 (1.82, 3.61)
PFNA, ng/ml	0.71 (0.53, 0.97)	1.07 (0.74, 1.59)	0.87 (0.62, 1.28)
PFDA, ng/ml	0.31 (0.24, 0.04)	0.43 (0.31, 0.62)	0.33 (0.24, 0.47)
PFUnDA, ng/ml	0.28 (0.22, 0.37)	0.44 (0.33, 0.63)	0.36 (0.26, 0.53)
PFHxS, ng/ml	2.09 (1.61, 3.46)	3.24 (2.05, 6.26)	2.87 (1.8, 10.46)
L-PFOS, ng/ml	13.31 (10.09, 17.89)	12.52 (7.96, 19.19)	7.57 (5.34, 11.48)
PFOSA, ng/ml	0.11 (0.08, 0.17)	0.07 (0.04, 0.13)	0.01 (0.02, 0.05)
**Kidney function**	**Mean (+/−SD)**	**Mean (+/−SD)**	**Mean (+/−SD)**
GFR, ml/min/1.73 m^2^	86.33 (13.67)	70.82 (13.67)	62.16 (14.67)

### Cross-sectional associations between PFAS concentrations and eGFR

**Fig. 1:**
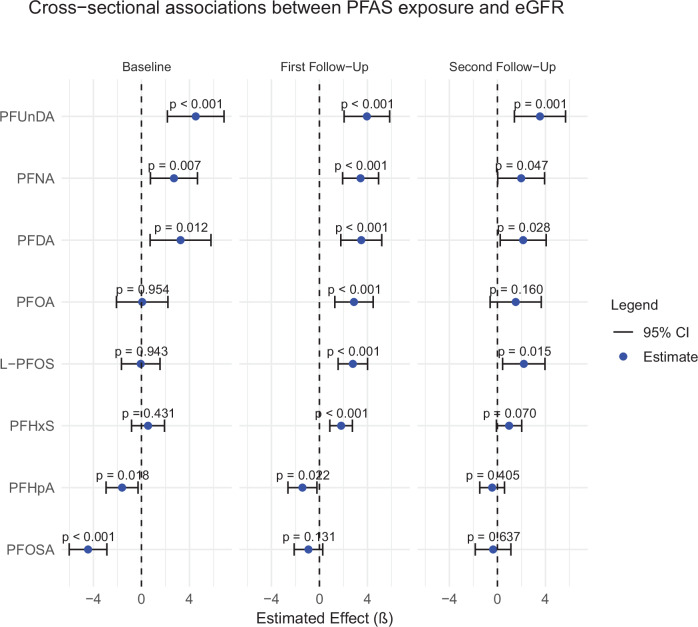
Cross-sectional association between PFAS and eGFR at ages 70, 75, and 80. Linear regression models were used to assess the relationship between eight ln-transformed PFAS and eGFR, adjusting for BMI, smoking, cholesterol (HDL and LDL), triglycerides, glucose, and statin use. BMI Body Mass Index, HDL High-Density Lipoprotein, LDL Low-Density Lipoprotein cholesterol, β Beta coefficient, CI confidence interval.

The cross-sectional analysis of PFAS and eGFR at the three time points revealed that most PFAS were significantly and positively associated with eGFR at each time point (Fig. 1). In contrast, PFHpA and PFOSA were the only compounds that showed an inverse association with eGFR. The results were largely consistent across the three timepoints.

### Associations between baseline PFAS concentrations and eGFR over time

**Fig. 2:**
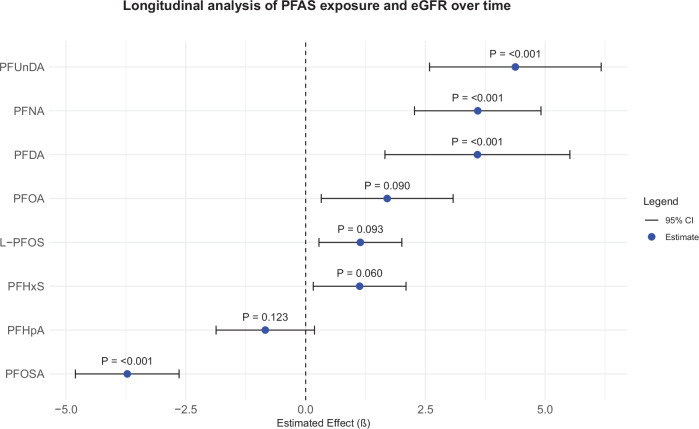
Longitudinal associations between PFAS concentrations and eGFR over time. Main effects (β) and 95% confidence intervals (CI) represent the overall association of PFAS with eGFR, while interaction effects indicate modification of this relationship over time as shown in Table 3. Models were adjusted for age (time), BMI, smoking, HDL- and LDL-cholesterol, triglycerides, glucose, and statin use. BMI Body Mass Index, HDL High-Density Lipoprotein and LDL Low-Density Lipoprotein, cholesterol, β Beta coefficient, CI confidence interval.

Utilizing the non-linear natural splines approach, we modeled the time-varying effects of baseline PFAS on eGFR to capture dynamic changes in the association over time following adjustment for age, smoking, BMI, cholesterol, triglycerides, glucose, and statin use (Table 3). Our findings indicated that for the majority of baseline PFAS, there was a positive association with eGFR (Fig. 2), while the time varying interaction effects suggested that this relationship attenuated over time. In contrast, baseline PFHpA and PFOSA demonstrated an inverse association with eGFR, with interaction effects suggesting that this association became stronger over time. However, the confidence intervals for both the interaction effects were wide, indicating considerable uncertainty in the estimates. As a result, while the general trend suggests a positive association between PFAS and eGFR, the time-varying effects remain weak and should be interpreted with caution.

**Table 3:** Associations between the baseline PFAS concentrations and the change in eGFR over time following adjustment for age (time), BMI, smoking, HDL- and LDL-cholesterol, triglycerides, glucose, and statin use.

PFAS	Main effect (β), 95% CI	Interaction effect (β), 95% CI
PFHpA	−0.842 (−1.869, 0.184)	0.357 (−1.983, 2.699)
PFHxS	1.128 (0.159, 2.096)	0.029 (−1.979, 2.038)
PFOA	1.702 (0.326, 3.078)	0.446 (−2.628, 3.521)
PFNA	3.593 (2.274, 4.912)	−1.891 (−4.818, 1.037)
PFDA	3.586 (1.656, 5.516)	−2.125 (−6.100, 1.852)
PFOSA	−3.722 (−4.805, −2.640)	5.805 (3.334, 8.277)
PFUnDA	4.377 (2.586, 6.169)	−3.090 (−6.912, 0.732)
L-PFOS	1.143 (0.278, 2.008)	0.625 (−1.377, 2.627)

*BMI* Body Mass Index, *HDL* High-Density Lipoprotein, *LDL* Low-Density Lipoprotein cholesterol, *β* Beta coefficient, *CI* confidence interval.

The exclusion of covariates BMI, HDL- and LDL-cholesterol did not alter the associations (Supplementary Table 2). To assess the potential for reverse causation, we modeled the associations between baseline eGFR and the change in PFAS concentrations over time, incorporating the same covariates as in the main model. The results demonstrated a minimal tendency for reverse causation, supporting the directionality of the PFAS vs eGFR relationship (Supplementary Table 3). Finally, when we restricted the analyses to individuals who participated in all three investigations (complete case analysis), the results were consistent with the main analyses (Supplementary Table 4).

## Discussion

### Main findings

The purpose of this study was to investigate the cross-sectional and longitudinal associations between PFAS and eGFR. The study included three repeated measurements of eight different PFAS measured in plasma and eGFR in an elderly general population. The cross-sectional relationship between PFAS concentrations and eGFR at baseline, first follow-up, and second follow-up varied by compound, with three PFAS showing positive associations, two showing inverse associations, and three displaying no significant relationship. The longitudinal analysis largely confirmed the findings from the cross-sectional analysis, showing that three PFAS (PFNA, PFDA, and PFUnDA) were positively associated with eGFR while findings for PFOA, PFHxS, and L-PFOS displayed a positive trend albeit non-significant. Only PFHpA and PFOSA displayed inverse associations. In general, while our findings suggest a general positive association between PFAS and eGFR, the wide confidence intervals indicate considerable uncertainty in the time-varying interaction effects.

### Comparison with previous literature

Several previous cross-sectional and longitudinal studies have investigated the associations of PFAS and eGFR in adult and pediatric populations (Table 4). The challenge in studying PFAS and kidney function lies in the potential for unadjusted confounding variables to introduce instability in the direction of associations, as well as the risk of reverse causation, since a decrease in GFR may reasonably lead to elevated serum concentrations of PFAS. Consequently, cross-sectional studies examining the relationship between serum PFAS levels and eGFR or kidney function emphasize that causality remains uncertain due to the limitations of this study design [13, 14, 28–31].

**Table 4:** An overview of longitudinal and cross-sectional studies investigating the association between PFAS and eGFR.

Author	Population	PFAS included	Methods	Main results	Conclusion
Longitudinal studies					
Li et al. [44]	967 diabetes patients from the Dongfeng-Tongji cohort, China.	PFOA, PFOS	Multivariable logistic regression. Least Absolute Shrinkage and Selection Operator (LASSO). Restricted Cubic Spline Regression (RCS).	Statistically significant inverse association was observed between PFOS and the risk of CKD (eGFR < 60 mL/min/1.73 m^2^).	Caution must be exerted interpreting the findings given the complexity of the relationship between kidney function and serum PFAS concentrations.
Lin et al. [15]	875 prediabetics from various medical centers, USA.	PFOA, PFOS, PFOSA, PFNA, PFHxS, Me-PFOSA^a^, Et-PFOSA^b^	Generalized linear mixed models. Linear regression.	PFOS and PFOA isomers were inversely associated with eGFR. For PFASs as a mixture, every one-quartile increase was associated with 2.26-unit decrease in eGFR.	Baseline plasma PFAS was inversely associated with prospective measures of eGFR. Higher baseline plasma concentrations were associated with lower eGFR over 14 years.
Blake et al. [10]	210 participants, Fernald Community Cohort, USA.	PFOA, PFOS, PFOSA, PFNA, PFHxS, PFDeA, Me-PFOSA^a^, Et-PFOSA^b^	Linear mixed effects models.	Statistically significant negative association between eGFR and PFNA, PFDeA, PFHxS.	Associations between PFAS and eGFR were generally negative, although differences by PFAS congener was observed.
Cross-sectional studies					
Dhingra et al. [13]	9192 women (age 30–65) from the C8 health project, West Virginia, USA.	PFOA	Linear regression. Pharmacokinetic and environmental prediction model.	Cross-sectional analysis showed a negative association between PFOA and eGFR. Modeled PFOA or cumulative exposure did not find any association.	The negative association between eGFR and measured PFOA is consistent with an increase in serum PFOA that might be expected to result from decreased kidney clearance of PFOA.
Moon et al. [14]	Adults from the National Health and Nutrition Examination Surveys, USA.	PFOA, PFOS, PFHxS, PFNA	Multivariable linear regression. Generalized additive model. Regression discontinuity model. Directed acyclic graphs.	All of the included PFAS was inversely associated with eGFR.	The possibility of reverse causation that increased serum PFAS concentration is the consequence of reduced eGFR and not the cause was low. Further longitudinal epidemiological and toxicological studies are needed.
Liang et al. [30]	1700 adults from the National Health and Nutrition Examination Surveys, USA.	PFOA, PFOS, PFNA, PFDA, PFDeA^c^, PFHxS	Multivariable linear regression. Bayesian kernel machine regression.	Higher levels of PFOS and PFHxS were significantly associated with lower eGFR. Joint effects of multiple PFAS on eGFR.	Prospective studies need to explore the joint effects further, and the biological mechanisms behind the associations needs to be further confirmed.
Kataria et al. [29]	1960 adolescents (12–19 years) from the National Health and Nutrition Examination Surveys, USA.	PFOA, PFOS, PFNA, PFHxS	Linear regression, univariable, bivariable and multivariable.	Adolescents in the highest PFOA and PFOS quartile had a lower eGFR compared to the lowest quartile.	Higher levels of PFAS are associated with a reduction in kidney function in otherwise healthy adolescents. Reverse causation and residual confounding could explain the results.
Shankar et al. [28]	4587 adults from the National Health and Nutritional Examination Survey, USA.	PFOA, PFOS	Multivariable linear regression.	Serum levels of PFOA and PFOS were positively associated with CKD (GFR less than 60 mL/minute/1.73 m2.	Elevated PFAS levels are associated with CKD.
Watkins et al. [45]	9660 children (1–18 years) from the C8 health project, West Virginia, USA.	PFOA, PFOS, PFNA, PFHxS	Linear regression. Pharmacokinetic and environmental prediction model.	An interquartile range increase in measured PFOA was associated with a decreased eGFR.	The findings of the study suggest that the cross-sectional association between eGFR and s-PFOA observed may be a consequence, and not a cause of decreased kidney function.
Conway et al. [31]	53,650 adults from the C8 health project, West Virginia, USA.	PFHxS, PFOS, PFNA, PFOA	Linear regression. Chi2-test. Logistic regression.	Each PFAS was positively associated with eGFR among those with CKD or anemia. These relationships were more pronounced along those with diabetes.	PFAS are inversely associated with kidney function in CKD and diabetes, with a stronger relation observed when anemia is present.

^a^N-Methylperfluorooctanesulfonamidoacetic acid.

^b^N-ethyl-perfluorooctane sulfonamido acetic acid.

^c^Perfluorodecanoic acid.

From this perspective, longitudinal studies may offer a more robust approach that is less susceptible to reverse causation. In our 10-year study, we observed an positive association between changes in plasma concentrations of PFNA, PFUnDA, and PFDA and eGFR while PFOA, PFHxS, L-PFOS showed non-significant results and PFHpA and PFOSA showed inverse associations. These mixed findings align with results from a longitudinal investigation in the Fernald Community Cohort, which assessed serum PFAS levels and eGFR over an 18-year period [10]. In that study, adjusted repeated measures models showed an interquartile range (IQR) increase in PFNA, PFHxS, and PFDA levels were associated with a decline in eGFR, while Me-PFOSA and Et-PFOSA demonstrated a positive association with eGFR [10]. Together, these studies suggest that the association between PFAS and eGFR is mixed and may not be consistent across all PFAS congeners.

Li et al. found that serum PFOS and PFOA concentrations were inversely associated with incident CKD risk among patients with diabetes (*n* = 967) and suggested that the high oxygen-carrying capacity of PFAS might mitigate the intrarenal hypoxia linked to diabetes. However, the authors emphasized that these findings should be interpreted cautiously, as no prior longitudinal studies have explored this relationship specifically in diabetic populations [32].

Supporting findings were reported in a large cross-sectional study based on the C8 Health Project where a positive relationship between PFAS and eGFR among individuals with CKD or anemia was reported [31]. Interestingly, in the absence of both CKD and anemia, PFAS were inversely associated with eGFR. The study suggested that PFAS may contribute to preserving renal function in CKD, potentially due to their oxygen-carrying properties that counteract hypoxia-induced progression, particularly in diabetic populations [31].

In a study on pre-diabetic adults, an inverse association between concentrations of six PFAS chemicals as a mixture and repeated measures of eGFR over 14 years was reported [15]. Statistically significant inverse associations between baseline PFOA and PFOS and change in GFR was also reported [15].

Uncertainties in the observations of PFAS may exist because they are based on observations in groups with differing exposure patterns. The concentrations measured in this study were lower compared to the concentrations in some previous studies [10, 15, 31, 32]. We only observed statistically significant inverse associations between PFOSA and PFHpA exposure and eGFR. However, PFOSA and PFHpA are generally low in abundance. Moreover, both compounds exhibited a substantial decreasing trend in our cohort, with PFOSA declining by 75% and PFHpA by 50% over 10 years. Given these factors, the potential public health implications of the relationship PFOSA and PFHpA exposure with eGFR appear limited, and caution is warranted when interpreting their potential health effects.

While we did not find any apparent inverse association to eGFR with PFAS chain length in our study, the PFAS most strongly associated with eGFR were long-chain compounds, particularly those with carboxylic acid functional groups. However, these associations should be interpreted carefully as we only include a limited number of congeners, and these do not necessarily translate to other PFAS.

Nephrotoxicity in humans has been hypothesized to occur when PFAS is reabsorbed across the renal tubules. Due to reabsorption of PFAS concomitant to continuous exposure, the potentially harmful effects on the kidneys may be ongoing [8, 10]. Several renal transporters have been shown to transport PFAS with differing affinity depending on the specific PFAS [7]. Adding to the complexity, kidney disease status may affect renal transporter expression levels and thereby impact the renal PFAS elimination [33]. Interestingly, a cross-sectional study by Jain et al. demonstrated an inverted U-shaped relation between PFAS and eGFR, suggesting that as kidney function declines from Stage 1, PFAS levels tend to rise initially and then decrease in advanced stages, resulting in a gradual inverted U-shaped pattern [34]. These findings could imply that the balance between actively mediated secretion and reabsorption may differ depending on the stage of kidney function.

The potential mechanisms by which PFAS may harm kidney function have been investigated but are not fully elucidated. The primary point of PFAS injury within the renal parenchyma remains unclear, with toxicological research indicating tubular histologic and cellular changes [8]. Experimental studies support that the renal elimination of PFAS is influenced by sex, species, and chain length, with organic anion transport proteins playing a key role in their reabsorption [35, 36]. Most PFAS, except perfluorobutane sulfonate (PFBS), have been shown to be substrates for the human organic anion transporter 4 (OAT4) in the kidney [7]. Histologically, studies on rodents have demonstrated that PFAS exposure can lead to tubular epithelial hyperplasia, apoptosis, increased kidney weight, and cortical and medullary congestion [8, 37]. PFOS exposure in rodents induced nephrotoxicity through oxidative stress and upregulation of Cx43 leading to tubular epithelial cell apoptosis [38]. Another important PFAS target is the nuclear receptor peroxisome proliferator alpha (PPAR-alpha) which is highly expressed in the proximal renal tubules and involved in metabolic signaling, specifically in fatty acid catabolism, peroxisome proliferation and inflammation [39].

While there exists a gap between experimental and epidemiological studies, new approaches and markers utilizing ”omics” technologies are emerging. These approaches might offer a more comprehensive understanding of the connections between exposures and outcomes [40]. PFAS exposure is associated with lower levels of the antioxidant protein α-klotho in individuals with normal kidney function, though reversed (positive) associations appear in more advanced kidney disease stages [41]. Using metabolomics, higher exposure to PFAS is associated with biomarkers related to kidney damage, oxidative stress and fatty-acid oxidation disruption when comparing occupational workers to a general population [42]. More recently, higher PFAS burden was associated with reduced kidney function in a longitudinal study of young adults from the Children’s Health Study, with changes in gut bacteria (such as reduced Lachnospiraceae) and specific metabolites mediating this relationship [43]. In addition to bridging the gap in previous study designs, these findings provide further insight into the potential mechanistic pathways.

### Strengths and limitations

The strengths of our study included the longitudinal design which incorporated repeated measures for plasma PFAS and GFR over a fairly long time. This design increases the certainty for observing causal relationships while lowering the risk of reverse causation. Yet, due to the nature of observational studies, causality can never be completely ascertained, and reverse causation cannot be refuted. Further, longitudinal studies inevitably encounter missing data given the multiple data collection points, which increases the likelihood of patient attrition and non-response. We therefore implemented both cross-sectional analyses and non-linear mixed effects models as a way to ensure consistency across the dataset.

The present study also had limitations. The use of an elderly population may limit the generalizability to other populations of different ages. Although we adjusted for several factors, including those related to chronic kidney disease, there is a possibility that other aspects associated with aging may affect kidney function. Finally, we only relied on one marker to assess kidney function. Although eGFR is used in several similar studies and provides comparable results, there may be other aspects of kidney function, such as albuminuria, affected by PFAS that have not been evaluated.

## Conclusion

Utilizing both cross-sectional and longitudinal approaches, our findings demonstrated a positive association between plasma PFNA, PFDA, and PFUnDA concentrations and eGFR, although not all compounds exhibited this pattern. Conversely, the plasma concentrations of PFOA, PFHxS, and L-PFOS showed positive albeit not significant associations with eGFR while only PFHpA and PFOSA were found to be inversely associated with eGFR. Considering the low abundance and the substantial decline in PFOSA and PFHpA levels in our and other populations, their potential public health impact appears to be limited. In conclusion, the findings of our study highlight the complexity of the relationship between PFAS concentrations and kidney function.

## Supplementary information

Supporting Information

## Data Availability

The data supporting the findings of the study are not publicly accessible and will be made accessible upon reasonable requested to the corresponding author.
