# Supplementary material for: Longitudinal and cross-sectional analysis of perfluoroalkyl substances and kidney function
Source: J Expo Sci Environ Epidemiol. 2025 Jun 9;35(6):1041–9. doi: 10.1038/s41370-025-00785-z (PMC12583146; doi:10.1038/s41370-025-00785-z)
Supplement: Supplementary file 1 — Supporting Information [file 41370_2025_785_MOESM1_ESM.docx]

**Supplementary Information for**

**Longitudinal and cross-sectional analysis of perfluoroalkyl substances and kidney function**

Eklund, A^1^., Taj, T^2^., Dunder L^23^., Lind, PM ^3^., Lind, L^34^. Salihovic, S*^1^.

^1^School of Medical Sciences, Faculty of Medicine and Health, Örebro University, Örebro, Sweden; ^2^Clinical Epidemiology and Biostatistics, School of Medical Sciences, Faculty of Medicine and Health, Örebro University, Örebro, Sweden; ^3^ Department of Medical Sciences, Occupational and Environmental Medicine, Uppsala University, Uppsala, Sweden;^4^ Department of Medical Sciences, Uppsala University, Uppsala, Sweden

***Corresponding author:**

Samira Salihovic, PhD

School of Medical Sciences, Faculty of Medicine and Health, Örebro University, Örebro, Sweden., 701 82 Örebro, Sweden.

Telephone: +46 19303226.

E-mail: [samira.salihovic@oru.se](mailto:samira.salihovic@oru.se)

**Table of Contents**

**Supplementary Table 1.** Linear vs. spline models for the longitudinal association of plasma PFAS concentration with eGFR

**Supplementary Figure 1**. Diacyclic graph (DAG) of the hypothesized causal relationships between PFAS exposure and eGFR. Abbreviations: BMI, body mass index; estimated glomerular filtration rate, eGFR, high-density lipoprotein cholesterol, HDL; low-density lipoprotein cholesterol, LDL; perfluoroalkyl substances (PFAS).

**Supplementary Table 2.** Associations between baseline PFAS concentrations and eGFR over time without covariate adjustment for BMI, HDL- and LDL-cholesterol from the models. All other covariates (age, triglycerides, smoking, glucose, and statin use) were retained.

**Supplementary Table 3.** Associations between baseline eGFR and PFAS concentrations over time to assess potential for reverse causation.

**Supplementary Table 4.** Associations between the baseline PFAS concentrations and the change in eGFR over time including only individuals who participated in all three investigations (complete case analysis).

**Supplementary Table 1.** Linear vs. spline models for the longitudinal association of plasma PFAS concentration with eGFR

| PFAS | Linear AIC | Spline AIC |
| --- | --- | --- |
| PFHpA | 24502,99 | 24467,92 |
| PFHxS | 24509,03 | 24456,69 |
| PFOA | 24508,45 | 24450,5 |
| PFNA | 24491,98 | 24422,94 |
| PFDA | 24507,29 | 24444,59 |
| PFOSA | 24489,42 | 24449,7 |
| PFUnDA | 24505,61 | 24435,32 |
| L-PFOS | 24498,27 | 24443,56 |

Note: AIC (Akaike Information Criterion) values are presented for both linear and spline models. All models were adjusted for age (time), BMI, smoking, HDL- and LDL-cholesterol, triglycerides, glucose, and statin use.


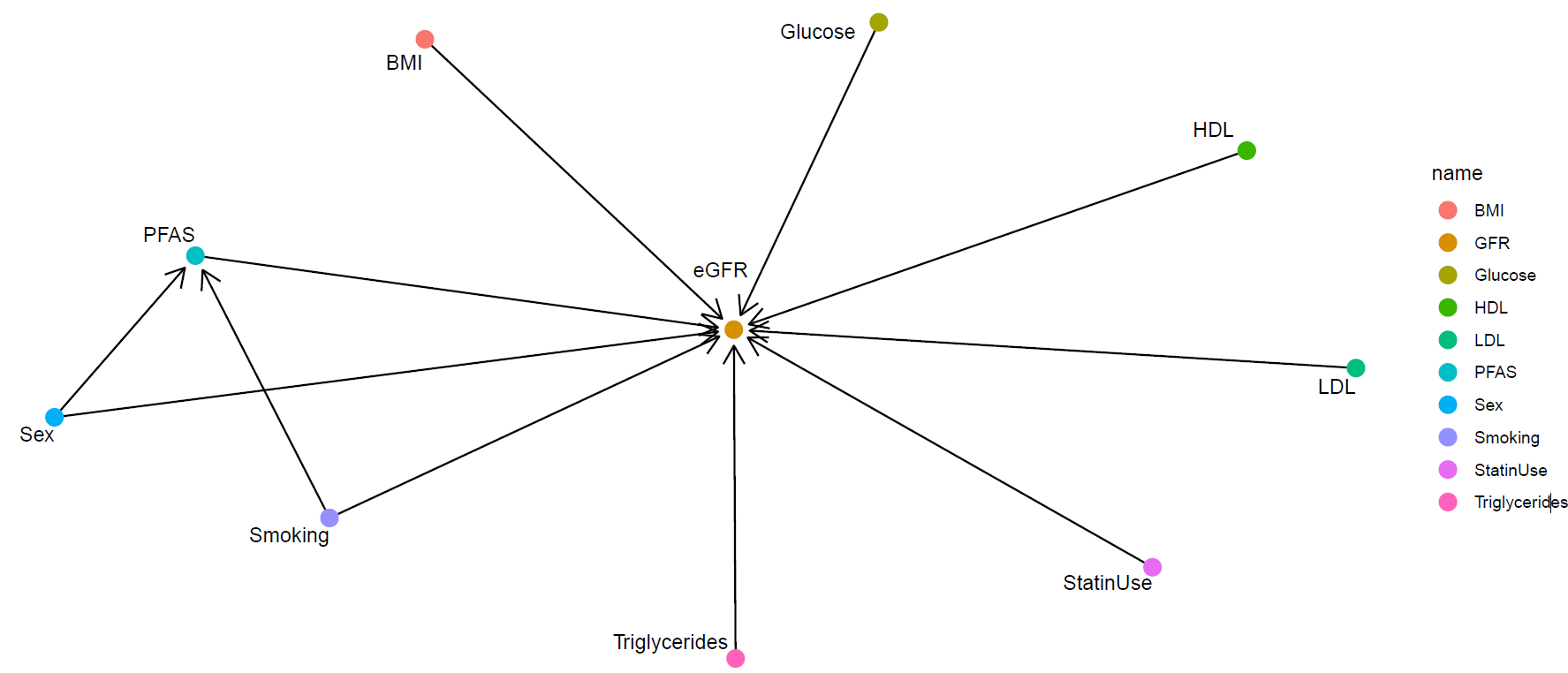


**Supplementary Figure 1**. Diacyclic graph (DAG) of the hypothesized causal relationships between PFAS exposure and eGFR. Abbreviations: BMI, body mass index; estimated glomerular filtration rate, eGFR, high-density lipoprotein cholesterol, HDL; low-density lipoprotein cholesterol, LDL; perfluoroalkyl substances (PFAS).

**Supplementary Table 2.** Associations between baseline PFAS concentrations and eGFR over time without covariate adjustment for BMI, HDL- and LDL-cholesterol from the models. All other covariates (age, triglycerides, smoking, glucose, and statin use) were retained.

| PFAS | Main effect (β), 95% CI | Interaction effect (β), 95% CI |
| --- | --- | --- |
| PFHpA | -0.657 (-1.690, 0.375) | 0.165 (-2.191, 2.521) |
| PFHxS | 1.287 (0.313, 2.261) | -0.025 (-2.046, 1.997) |
| PFOA | 2.036 (0.654, 3.417) | 0.159 (-2.933, 3.252) |
| PFNA | 3.915 (2.592, 5.237) | -2.103 (-5.045, 0.841) |
| PFDA | 4.286 (2.360, 6.213) | -2.664 (-6.657, 1.329) |
| PFOSA | -3.552 (-4.642, -2.462) | 5.630 (3.145, 8.116) |
| PFUnDA | 5.081 (3.300, 6.864) | -3.376 (-7.215, 0.463) |
| L-PFOS | 1.310 (0.441, 2.180) | 0.542 (-1.472, 2.557) |

**Supplementary Table 3.** Associations between baseline eGFR and PFAS concentrations over time to assess potential for reverse causation.

| PFAS | Main effect (β), 95% CI | Interaction effect (β), 95% CI |
| --- | --- | --- |
| PFHpA | -0.002 (-0.005, 0.000) | 0.001 (-0.001, 0.002) |
| PFHxS | 0.003 (0.000, 0.006) | 0.000 (-0.002, 0.001) |
| PFOA | 0.002 (0.000, 0.003) | 0.000 (-0.001, 0.001) |
| PFNA | 0.003 (0.002, 0.005) | -0.001 (-0.002, 0.000) |
| PFDA | 0.002 (0.000, 0.003) | -0.001 (-0.002, 0.000) |
| PFOSA | -0.005 (-0.006, -0.003) | 0.003 (0.002, 0.004) |
| PFUnDA | 0.002 (0.001, 0.004) | -0.001 (-0.002, 0.000) |
| L-PFOS | 0.004 (0.001, 0.006) | 0.000 (-0.002, 0.002) |

Note: All models were adjusted for age (time), BMI, smoking, HDL- and LDL-cholesterol, triglycerides, glucose, and statin use.

**Supplementary Table 4.** Associations between the baseline PFAS concentrations and the change in eGFR over time including only individuals who participated in all three investigations (complete case analysis).

| PFAS | Main effect (β), 95% CI | Interaction effect (β), 95% CI |
| --- | --- | --- |
| PFHpA | -0.050 (-1.907, 1.810) | -1.639 (-5.428, 2.138) |
| PFHxS | 1.047 (-1.091, 3.183) | 0.234 (-3.841, 4.319) |
| PFOA | 3.464 (0.233, 6.697) | -2.121 (-8.384, 4.131) |
| PFNA | 2.233 (-0.577, 5.047) | -0.498 (-5.943, 4.939) |
| PFDA | 2.120 (-1.462, 5.703) | -0.759 (-7.609, 6.089) |
| PFOSA | -2.684 (-4.598, -0.766) | 3.516 (-0.414, 7.433) |
| PFUnDA | 0.456 (-2.748, 3.660) | 3.227 (-3.049, 9.506) |
| L-PFOS | 1.547 (-1.028, 4.124) | 1.251 (-3.614, 6.109) |

Note: All models were adjusted for age (time), BMI, smoking, HDL- and LDL-cholesterol, triglycerides, glucose, and statin use.
